# News and Events of the Week

**Published:** 1910-01-29

**Authors:** 


### News and Events of the Week.

Dr. C. B. Plowright, medical officer of health under the Freebridge Lynn Rural District Council, has resigned his appointment owing to failing health.

Dr. C. F. Fenton, for 14 years medical officer of health at Barking, on resigning his post, has received a handsome presentation f[illegible]rict Council and the Education Committee.

Sir William J. Sinclair, M.A., M.D., M.R.C.P., Professor of Obstetrics and Gynæcology, has been appointed Pro-Vice-Chancellor of Manchester University in place of Professor William Stirling, whose term has expired.

A decree of judicial separation was awarded in the High Court last week on the petition of Mrs. May Traill-Thompson, the wife of Dr. William Traill-Thompson, a practitioner at Wembley, on the ground of his cruelty.

A resolution protesting against any interference with the "Brown Dog" memorial was carried at Battersea Town Hall on Tuesday night. There was some opposition from a band of students, two of whom addressed the meeting.

Mr. Robert Thomas Gunn, F.R.C.S., M.B., of Hindhead, President of the Ophthalmic Society, late senior surgeon to the Moorfields Ophthalmic Hospital, who assisted in preparing the results of the *Challenger* Expedition, has left estate valued at £28,193 gross, and personalty £25,563 net.

At the recent annual meeting of the Midwives' Institute much regret was caused by the announcement of the death of Dr. Stanley Atkinson, who had represented this body on the Central Midwives Board for the past three years. Dr. Macrory (Inspector of Midwives for London) was one of the newly-elected vice-presidents.

The prize of £50 awarded annually by Sir John Craggs, M.V.O., to a past or present student of the London School of Tropical Medicine who during the current year has made the most valuable contribution to tropical medicine has this year been awarded to Dr. G. A. Turner, of Johannesburg, for "An Account of some Helminthes occurring among the South African Natives."

Radium banks already exist in Paris, Berlin, and New York. It is now reported that one is to be established in London, and will be opened in the course of the year in the Cavendish Square district. Its object will be to amass radium and lend it, on deposit of a banker's guarantee, to medical and scientific men, who may need it for treatment of cancer and other cases. Its price now is about £20 a milligram.

Among the schemes for providing employment under consideration by the Central (Unemployed) Body are the formation of a lake in Southwark Park, 240 feet long and 80 feet wide, similar to that constructed at Tooting Common four years ago; a recreation ground at Sawley Road, Hammersmith; the levelling of school sites; the widening and improvement of Tooting Bec Road; and the laying-out of playing fields at Dulwich for the Borough Polytechnic Institute.

Dr. W. H. Beach, medical superintendent of the Yarmouth Isolation Hospital, medical officer of health and port medical officer, has resigned his posts.

The Chinese department of the University Library at Cambridge has recently received a gift of over 100 volumes from Dr. F. Sanger, of St. John's College, who, after taking his M.D., went to China as a medical missionary. Among the most important of the books may be mentioned various editions of poetical remains, anthologies, etc., dated 1670, 1699, 1703, 1709, 1711, and 1772.

The annual meeting of the British Medical Association will be held in London from July 26 to 29 next, under the presidency of Mr. H. T. Butlin, P.R.C.S. One of the most interesting of the 21 sections of scientific work will be the public health section, of which Sir Walter Foster, M.P., is president; while a special section will be devoted to radiology and medical electricity. Questions particularly concerning the Navy, Army, and Territorial Forces will also form a special section. The Corporation of the City of London will give a reception in the Guildhall in honour of the association during the week of its meetings in London.

An action arising out of the sale of a medical practice came before Mr. Justice Jelf at Ipswich last Saturday. Mr. Oscar Jacobsen, medical practitioner of Orford, in Lincolnshire, brought an action against Mr. Leslie Fletcher, practitioner at Hartest, near Bury St. Edmunds, to recover damages for fraudulent misrepresentation in the sale of a practice, and a declaration that the plaintiff was entitled to £100 now in Court. The defendant, it was stated, wishing to sell his practice said its income was over £100, and that certain public appointments would, almost certainly, be transferred. In fact these passed to a third party, but the action was settled by the plaintiff withdrawing the charges of misrepresentation, by the defendant paying the plaintiff's taxed costs, and by the plaintiff receiving £50 of the £100—which was the sum he had deposited with an agent before the action, on agreeing to buy the practice.

Christian Science was again in the courts when an inquest was held at Chelsea, on January 19, on the death of Captain John Robert Donne, formerly in the Dragoon Guards and a Christian Scientist, who died at Chelsea on the 15th inst. His wife said he had suffered from locomotor ataxy, but had not seen a doctor for eight years, as he was a Christian Scientist. Mrs. Jessie Braithwaite, of Regent's Park, said she was a Christian Science practitioner, and had attended Captain Donne for seven years. Her treatment was a form of prayer. She was paid one guinea a week, or 4s. a treatment. Dr. Spilsbury, pathologist at St. Mary's Hospital, said a post-mortem examination showed the cause of death was heart failure set up by degeneration of the spinal cord and muscles. If Captain Donne had been under medical treatment during the whole of his illness his life might have been prolonged for another year. The Coroner, in summing up, said the case differed materially from many previous cases, since the relatives were perfectly willing that Captain Donne should see a doctor. It was a question of free agency, and it was clear the man, except on one occasion, expressed no desire to see a doctor. Verdict: "Death from natural causes," with an expression of regret that no medical man was called in.

